# Case Report: Positioning head tilt observed in six dogs with meningoencephalitis of unknown origin

**DOI:** 10.3389/fvets.2026.1709307

**Published:** 2026-02-23

**Authors:** Shinji Tamura, Koen M. Santifort, Takayuki Kuwabara, Yuya Nakamoto, Yumiko Tamura

**Affiliations:** 1 Tamura Animal Clinic, Hiroshima, Japan; 2 IVC Evidensia Referral Hospital Arnhem, Arnhem, Netherlands; 3 IVC Evidensia Referral Hospital Hart van Brabant, Waalwijk, Netherlands; 4 Kuwabara Animal Hospital, Maebashi, Japan; 5 Neuro Vets Animal Neurology Clinic, Kyoto, Japan

**Keywords:** cerebellum, dog, meningoencephalitis of unknown origin, midbrain, positioning head tilt

## Abstract

Positioning head tilt (PHT) is a dynamic clinical neurological sign that is characterized by a head tilt to the opposite side of a voluntary lateral turn of the head. Based on recent publications, various etiologies are proposed for the occurrence of PHT in dogs and cats. One suggested cause is a lack of inhibitory input to the vestibular nuclei due to dysfunction of the cerebellar nodulus and uvula (NU). In that category, it has been reported in dogs with hypoplasia of the NU, dogs with lysosomal storage diseases, and in a dog with a cerebellar tumor. Other proposed causes of PHT include reduced input of either proprioceptive information from the spindles of cervical muscles or information about head movement in space from peripheral vestibular apparatus. As examples of the former, it has been observed in feline cases of hypokalemic myopathy and myasthenia gravis. As an example of the latter, it has been observed in a dog and four cats with bilateral peripheral vestibular dysfunction. In this study, we describe and discuss our observations of PHT in six dogs with meningoencephalitis of unknown origin (MUO). Although it was not possible to identify the causative lesion site in these dogs, the possibility of MUO causing the clinical sign of PHT in dogs is deemed to be clinically relevant.

## 1 Introduction

Positioning head tilt (PHT) is a dynamic clinical neurological sign that is characterized by a head tilt to the opposite side of a voluntary lateral turn of the head (1, 2). A head tilt is absent when the head is held stationary or when the animal is moving forward. In dogs, PHT has been documented as a clinical sign of disorders affecting the cerebellum, including four dogs with cerebellar hypoplasia [specifically of the nodulus and uvula (NU)] (1, 3), a dog with a tumor invading the NU (4–6), and nine dogs with lysosomal disease (affecting the central nervous system diffusely, including the cerebellum) (7). In those cases, PHT is thought to be caused by a lack of inhibitory input to the vestibular nuclei due to NU dysfunction. Additionally, PHT has been reported in 14 cats with hypokalemic myopathy (8) and 2 cats with myasthenia gravis (9). In these cases, PHT is proposed to be due to altered function of muscle spindles in the rectus and obliquus capitis muscles, disrupting their proprioceptive input to the NU (8, 9). PHT has also been reported in a dog and four cats with bilateral peripheral vestibular dysfunction (10). In those cases, transmission of information from peripheral vestibular apparatus to the NU regarding position and movement of the head in space is disrupted, hindering the NU’s ability to inhibit the excitation of rostral, medial, and caudal vestibular nuclei induced by head movement (8–10).

To set the appropriate tone in the postural muscles, the cerebellum must be informed about movements planned by higher motor systems (11). In addition to proprioceptive information from the muscle spindles of the cervical muscles and information of head position and movement in space from the vestibular apparatus, this is essential for the cerebellum to set an appropriate postural platform before the motor activity is initiated and to regulate and coordinate muscle activity throughout motor activity (11). Signals involved in extrapyramidal coordination of semiautomatic movements, posture, and locomotion originate from the cerebrum, thalamus, and midbrain and are conveyed to the olivary nuclei of the medulla oblongata. These nuclei project to the contralateral cerebellum. The cerebellum is in turn connected, via the deep cerebellar nuclei, to the contralateral extrapyramidal nuclei of the forebrain (cortex and basal nuclei) and brainstem (11). This loop of information streaming throughout multiple brain regions is complex but essential for adequate maintenance of posture, regulation of movement, and application of appropriate force of motor activity. In this context, we cautiously hypothesize that PHT, as a clinical neurological sign, may arise from additional disorders and neuroanatomical lesion sites beyond those hitherto described.

In this study, we describe six cases of dogs clinically diagnosed with meningoencephalitis of unknown origin (MUO) presenting with PHT. We include hypotheses on the mechanisms involved to result in PHT in these animals, although we recognize that no firm conclusion can be based on this limited case series due to limitations and involvement of multiple lesions in different brain regions.

## 2 Case description

### 2.1 Case 1

**Figure 1:**
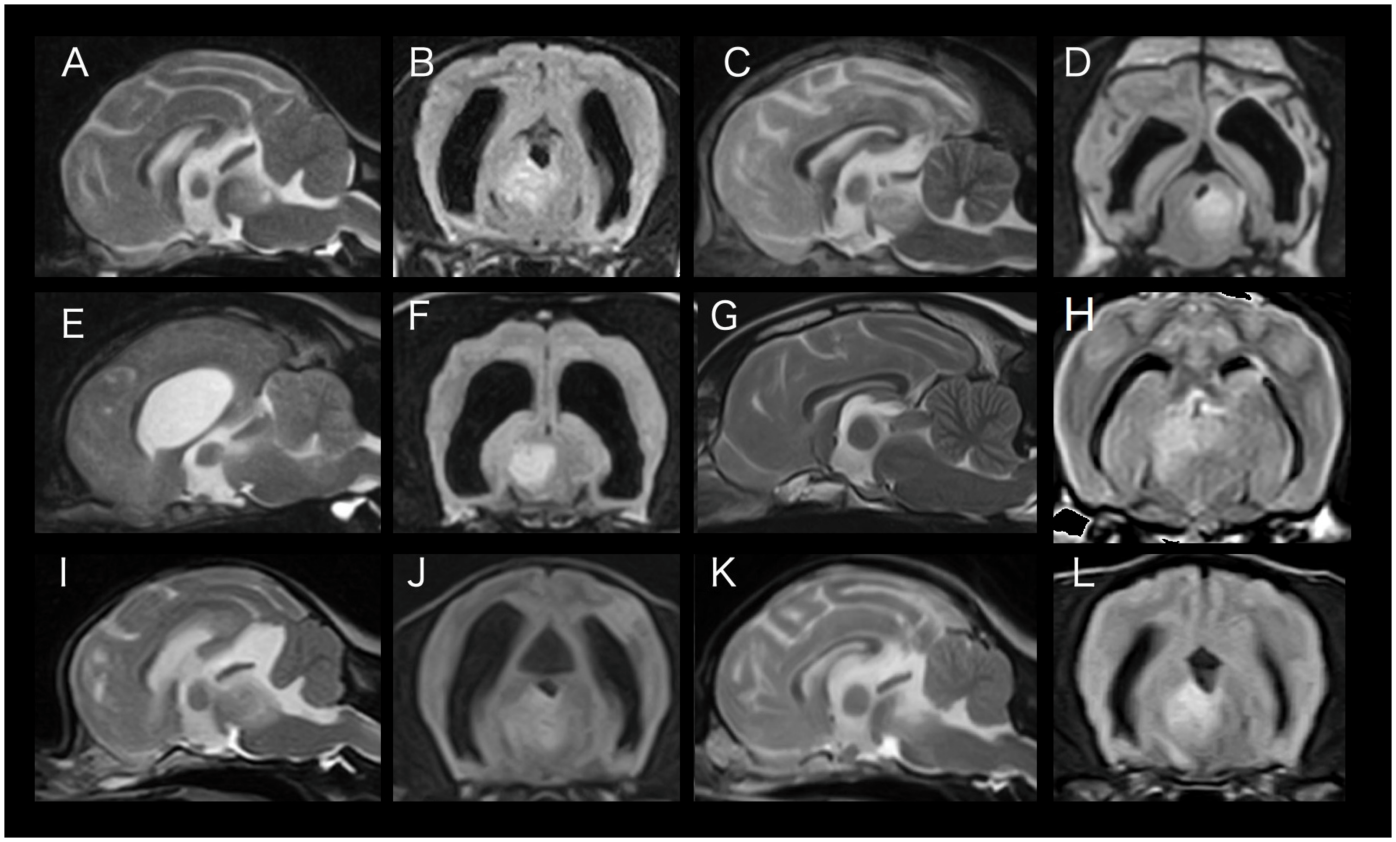
T2-weighted mid-sagittal magnetic resonance images (**A**: case 1, **C**: case 2, **E**: case 3, **G**: case 4, **I**: case 5, **K**: case 6) and fluid attenuated inversion recovery transverse images at the level of the midbrain (**B**: case 1, **D**: case 2, **F**: case 3, **H**: case 4, **J**: case 5, **L**: case 6). There were additional lesions elsewhere (multifocal) other than what is indicated here (see descriptions in the text per case). Lesions in the midbrain were deemed asymmetrical in all cases.

A 4-year and 10-month-old spayed female Chihuahua, was referred to Tamura Animal Clinic with a complaint characterized by the owner as an acute onset of “wobbly gait” one week earlier, which gradually worsened to persistent circling. Neurological examination revealed abnormalities including ataxia, hypermetria, a decreased left-sided menace response, and a head tilt to the left at rest and when the head was turned to the right but titled to the right when the head was turned to the left, consistent with PHT (Supplementary Video 1). A magnetic resonance imaging (MRI) study was performed (0.3 T Hitachi Airis II comfort). T2-weighted (T2W) and fluid attenuated inversion recovery (FLAIR) hyperintense lesions were observed in the right frontal lobe, right thalamus and bilateral midbrain (asymmetrically, right worse than left), and the right cerebellar hemisphere (Figures 1A,B). These lesions were hypointense on T1W images and did not show contrast enhancement. Cerebrospinal fluid (CSF) was not obtained due to financial constraints. MUO was suspected, and PHT as well as other clinical signs resolved after treatment with prednisolone (initially 2 mg/kg once daily and gradually decreased) and cytarabine (initially 50 mg/m^2^ twice daily for 2 days every 3 weeks for 3 times and every four weeks for four times and gradually decreased). The dog is still alive with a good quality of life with cytarabine twice daily for 2 days every 8 weeks after about 7 years since diagnosis.

### 2.2 Case 2

A 3-year-old female French Bulldog was presented for leaning to the right for 2 months and a chief complaint of difficulty standing. The dog had been diagnosed with MUO based on MRI findings of multifocal lesions (unspecified) at the age of 1 year and treated with prednisolone (initially 2 mg/kg once daily and gradually decreased) and phenobarbital (3 mg/kg twice daily) at the referring hospital. Neurological examination revealed abnormalities including non-ambulatory tetraparesis, abnormal mental status (sopor), bilaterally absent menace responses and pupillary light reflexes, rotatory nystagmus, and PHT (Supplementary Video 1). On MRI (0.3 T Hitachi Airis II comfort), T2W and FLAIR hyperintense lesions were observed in the left parietal, temporal, and occipital lobes (including suspected necrotic foci), right temporal lobe, left thalamus, and midbrain (bilateral, left worse than right) (Figures 1C,D). These lesions were hypointense on T1W images. Mild contrast enhancement was observed in the midbrain legion on post-contrast T1W images. A relapse of MUO was suspected. PHT disappeared and the patient was able to walk after increasing doses of prednisolone (initially 2 mg/kg once daily and gradually decreased). However, relapse occurred during tapering, followed by improvement after increasing the dose again. Finally, the patient escaped from home at the age of 4 years and died of heatstroke.

### 2.3 Case 3

A 1-year and 7-month-old male Pug was presented to Tamura Animal Clinic with a complaint of decreased level of consciousness for 5 days. Neurological examination revealed abnormalities including ataxia and proprioceptive deficits in all four limbs. Additionally, the dog showed a PHT (Supplementary Video 1). On MRI (0.3 T Hitachi Airis II comfort), T2W and FLAIR hyperintense lesions were observed in the right and left cerebrum, bilateral lesions from the thalamus to the midbrain more severe on the right side (with minor intraparenchymal contralateral hyperintensity and mass effect for right to the left side) and medulla oblongata (Figures 1E,F). These lesions were hypointense on T1W and did not show contrast enhancement. CSF was not collected due to the presence of a mild tentorial and transforaminal herniation. The dog was presumptively diagnosed with MUO and was treated with prednisolone (initially 2 mg/kg once daily and gradually decreased) and phenobarbital (3 mg/kg twice daily) at the referring hospital, and maintained a good quality of life. The dog died of hepatitis four years later.

### 2.4 Case 4

A 4-year and 9-month-old spayed female mixed breed dog was referred to IVC Evidensia Small Animal Referral Hospital in Arnhem with a chief complaint of acute progressive ataxia for 3 days. Neurological examination revealed an abnormal mental status (sopor), vestibular ataxia, proprioceptive deficits of the left thoracic and pelvic limbs, rotatory nystagmus, and tonic neck testing resulted in buckling of the limbs. The patient was considered to have PHT because the dog exhibited a head tilt to the left that worsened when the head was turned to the right, and the head was in a neutral position when the head was turned to the left (Supplementary Video 2). On MRI (1.5 T Canon Vantage Elan), T2W and FLAIR hyperintense lesions were observed from the thalamus to the midbrain bilaterally, more severe on the right side, and in the left cerebellar hemisphere, mainly in the white matter (Figures 1G,H). These lesions were isointense on T1W images. Contrast enhancement was not observed on post-contrast T1W. CSF was not collected due to the presence of a mild tentorial and cerebellar herniation. MUO was suspected, and signs, including PHT, resolved after 1 week of treatment with prednisone (initially 1 mg/kg twice daily and gradually decreased), but the patient relapsed at 7 weeks and remitted with increased doses. The patient was euthanized after the occurrence of epileptic seizure activity and MUO progression was diagnosed by MRI 13 weeks later.

### 2.5 Case 5

A 5-year and 9-month old spayed female Chihuahua was presented to Kuwabara Animal Hospital with a complaint of a head tilt to the left for a month, followed by the occurrence of circling and ataxia. Neurological examination revealed abnormalities including ataxia and proprioceptive deficits in all limbs, a mild head tilt to the left, horizontal nystagmus with fast phase to the right, anisocoria with left-sided miosis and PHT (Supplementary Video 3). On MRI (0.3 T Fuji Film, AIRIS Vent Plus), T2W and FLAIR hyperintense lesions were observed in the right parietal lobe and bilateral midbrain, more severe on the left side (Figures 1I,J). These lesions were slightly hypo- to isointense on T1W images. Mild contrast enhancement was observed in the parietal legion on post-contrast T1W images. An incidental arachnoid diverticulum (supracollicular fluid accumulation) and cervical spinal cord syringomyelia were diagnosed additionally. Pleocytosis with a mixed pattern of mononuclear cells and neutrophils (9/μL, reference range 0–5/μL) was revealed on CSF examination. CSF protein level was not examined. The dog was suspected to have MUO and was treated with prednisolone (initially 2 mg/kg once daily and gradually decreased), cytarabine (initially 100 mg/m^2^ continuous rate infusion for 24 h, then 50 mg/m^2^ bid for 2 days every 3 weeks for 3 times and spacing dosages out gradually) and cyclosporine (initially 5 mg/kg once daily and gradually decreased). PHT and other signs resolved shortly after initiation of treatment. The dog is still alive with a good quality of life after about 11 months since diagnosis.

### 2.6 Case 6

A 4-year and 9-month-old castrated male Chihuahua was presented to Neuro Vets Animal Neurology Clinic with a complaint of head tilt to both sides for 11 days and progressive ataxia. Neurological examination revealed abnormalities including ataxia and proprioceptive deficits in all limbs, a decreased menace response on the left side, a head turn to left at rest and PHT (Supplementary Video 3). On MRI (0.4 T Hitachi, APERTO Lucent), T2W and FLAIR hyperintense lesions were observed in the left temporal lobe, left thalamus, and bilateral midbrain, more severe on the left side (Figures 1K,L). These lesions were slightly hypointense on T1W images. Mild contrast enhancement was observed on post-contrast T1W images. Pleocytosis with a mixed pattern of mononuclear cells (12/μL) and elevated protein concentration (36.4 mg/dL) was revealed on CSF examination. The dog was presumptively diagnosed with MUO and was treated with prednisolone (initially 1.5 mg/kg twice daily and gradually decreased) and cytarabine (initially 100 mg/m^2^ continuous rate infusion for 24 h, then 50 mg/m^2^ twice daily for 2 days every 3 weeks for 3 times and spacing dosages out gradually). PHT resolved after treatment, but head turn persisted. The dog is still alive with a good quality of life after about 2 years since diagnosis.

## 3 Discussion

PHT was observed in all the dogs included in this study that were clinically diagnosed with MUO. The common overlapping feature was involvement of the midbrain, bilaterally and asymmetrically. Although we cannot exclude direct involvement of lesions in other sites, we hypothesize that PHT might be observed when input related to motor planning from this higher-level center to the cerebellum is bilaterally disrupted. However, we must stress that all dogs had multifocal lesions based on MRI studies and there is also the possibility of having lesions that could not be detected by MRI. Furthermore, none of the cases were definitively diagnosed with MUO based on histopathology and the extent of the lesions could not be ascertained on a microscopical level. Therefore, it is difficult to identifying the specific site of impairment responsible for PHT in each of these cases. Nonetheless, we judged our observation of PHT in dogs with MUO relevant to report, especially with the observation of midbrain involvement, as this feature can now be looked for specifically in future cases. Also, the other causes of PHT are markedly different in etiologies than this typically acute progressive and sometimes fatal disease. When PHT is observed in a dog, MUO should be included in the differential diagnosis list and prompt further investigation.

Lesions of the paracentral thalamus or the midbrain typically result in vestibular signs contralateral to the lesion (12–15). Cases 1, 3, 4, and 5 showed a head tilt and nystagmus to the side contralateral to the side where the midbrain lesion was more severe. We speculate that this could have been due to the difference in the degree of damage to pathways between the left and right parts of the thalamus and/or the midbrain.

Importantly, in cases 1, 4, 5 and 6, PHT disappeared after initiation of treatment, suggesting that PHT is reversible in dogs with MUO. Whether or not subsequent recurrence of this sign may be used as an indicator of MUO recurrence in clinical practice cannot be ascertained from our results, but is an interesting question and could possibly be of use in the follow-up of these patients.

Although MUO is a relatively common disease in dogs, the sign of PHT has not been recognized or reported in dogs with MUO. This is the first report of PHT in dogs clinically diagnosed with MUO. It may well be that PHT is often not reported in cases of MUO, as it is possibly not one of the most distressing clinical features or inadequately characterized merely as “a head tilt” in affected cases. Future studies including clinical feature descriptions of dogs with MUO should include as much detail as possible on the clinical signs. Possibly, specific clinical signs can point to involvement of certain brain structures and provide input for treatment considerations and follow-up plans.

There are numerous limitations to our study. These include most prominently the lack of CSF examination for cases 1–4, and the lack of histopathological examination in all cases (and therefore the lack of definitive diagnoses). Hence, we can only speculate on why PHT occurred in these dogs with multifocal brain lesions, even though the common denominator was involvement of the midbrain.

In conclusion, in addition to the previously reported sites of the NU, bilateral cervical muscle spindles and bilateral peripheral vestibular apparatus, lesions in other brain regions, possibly the (bilateral) midbrain and/or other centers that provide input to the cerebellum, may result in the clinical sign of PHT. MUO is relatively common disease and this particular sign might be helpful and valuable for clinicians to be aware of. In patients presented with a PHT, MUO should be included in the differential diagnosis list. With regard to pathophysiology of PHT in cases with lesions involving the midbrain, more focal pathology (e.g., vascular events or tumors), when identified, may yield more conclusive evidence for the midbrain as primary regulatory center involved in these cases of PHT. Obtaining histological confirmation and descriptions of lesion severity are also merited to strengthen the interpretation and clarify the relationship between identified brain lesions and PHT.

## Data Availability

The original contributions presented in the study are included in the article/Supplementary material, further inquiries can be directed to the corresponding author.

## Supplementary material

SUPPLEMENTARY VIDEO 1 Cases 1–3: positioning head tilt. Case 1: head tilt to the left at rest but to the right when the head is turned to the left. Case 2: head tilt to the opposite side of a voluntary lateral turn of the head. Case 3: head tilt to the left when at rest but tilt to the right when the head was turned to the left.

SUPPLEMENTARY VIDEO 2 Case 4: positioning head tilt. Head tilt to the left worsened when his head was turned to the right, and the head was in a neutral position when his head was turned to the left.

SUPPLEMENTARY VIDEO 3 Cases 5 and 6: positioning head tilt. Case 5: head tilt to the left at rest but to the right when the head is turned to the left. Case 6: head tilt to the opposite side of a voluntary lateral turn of the head.
